# Supplementary material for: Protection of Neuronal Diversity at the Expense of Neuronal Numbers during Nutrient Restriction in the Drosophila Visual System
Source: Cell Rep. 2013 Mar 28;3(3):587–94. doi: 10.1016/j.celrep.2013.02.006 (PMC3617362; doi:10.1016/j.celrep.2013.02.006)
Supplement: Document S1. Article plus Supplemental Information [file mmc1.pdf]

Cell Reports
Report

Protection of Neuronal Diversity at the Expense of Neuronal Numbers during Nutrient Restriction in the *Drosophila* Visual System

Elodie Lanet,[1] Alex P. Gould,[2] and Cédric Maurange[1,*]
[1]Aix-Marseille Université, CNRS UMR 7288, IBDM, Campus de Luminy Case 908, 13288 Marseille Cedex 9, France
[2]Division of Physiology and Metabolism, MRC National Institute for Medical Research, The Ridgeway, Mill Hill, London NW7 1AA, UK
*Correspondence: cedric.maurange@univ-amu.fr
http://dx.doi.org/10.1016/j.celrep.2013.02.006

SUMMARY

Systemic signals provided by nutrients and hormones are known to coordinate the growth and proliferation of different organs during development. However, within the brain, it is unclear how these signals influence neural progenitor divisions and neuronal diversity. Here, in the *Drosophila* visual system, we identify two developmental phases with different sensitivities to dietary nutrients. During early larval stages, nutrients regulate the size of the neural progenitor pool via insulin/PI3K/TOR-dependent symmetric neuroepithelial divisions. During late larval stages, neural proliferation becomes insensitive to dietary nutrients, and the steroid hormone ecdysone acts on Delta/Notch signaling to promote the switch from symmetric mitoses to asymmetric neurogenic divisions. This mechanism accounts for why sustained undernourishment during visual system development restricts neuronal numbers while protecting neuronal diversity. These studies reveal an adaptive mechanism that helps to retain a functional visual system over a range of different brain sizes in the face of suboptimal nutrition.

INTRODUCTION

In the mammalian fetus, as in insect larvae, systemic hormones such as insulin growth factors coordinate growth between developing organs in response to nutrients (Randhawa and Cohen, 2005). It is well documented that late-fetal nutrient deprivation in humans and other mammals can lead to sparing of the growth of the brain at the expense of other less critical organs (Gruenwald, 1963). However, when nutrient access is restricted from early fetal stages, the brain often exhibits isometric downscaling with the rest of the body. The mechanisms underlying these different adaptive responses and, more generally, the way in which nutrients influence overall neuronal number and brain size are poorly understood.

During early development, mammalian neural stem cells (NSCs) initially undergo a proliferative phase corresponding to a period of expansion through symmetric divisions, which forms a pseudostratified neuroepithelium (NE) (Farkas and Huttner, 2008; Götz and Huttner, 2005). Later, NE cells switch to a neurogenic phase, involving asymmetric divisions that generate a repertoire of neurons with different identities depending on their birth order (reviewed by Jacob et al., 2008; Okano and Temple, 2009). In mice, local signaling pathways have been shown to regulate proliferation of the NE and the switch to an asymmetric division mode (Aguirre et al., 2010; Falk et al., 2008; Sahara and O'Leary, 2009). Yet, it remains unclear if nutrients and systemic hormones regulate the number of both symmetric and asymmetric divisions to ensure that the correct number of each neuronal subtype is produced by the end of the development.

In *Drosophila*, neurogenesis occurs through the asymmetric division of NSCs called neuroblasts (NBs). In most regions of the CNS (central brain and ventral nerve cord), a fixed number of NBs is formed early during development, in the early embryo (Skeath and Thor, 2003). Most NBs undergo a period of quiescence at the end of embryogenesis and are awakened by feeding in the early larva, which stimulates glial-derived insulin-like peptides (Ilps), thus leading to NB activation of Insulin Receptor/Target of Rapamycin (InR/TOR) signaling (Chell and Brand, 2010; Sousa-Nunes et al., 2011). Concomitantly, organismal growth is promoted by systemic Ilps (Brogiolo et al., 2001). When the larva reaches a critical mass, the production of high levels of steroid hormone ecdysone antagonizes Ilps to terminate growth and trigger metamorphosis (Colombani et al., 2005; Layalle et al., 2008). While larval growth depends on the continuous supply of dietary nutrients (Ikeya et al., 2002), cycling NBs in late larvae can sustain growth and proliferation independently of dietary nutrients (Britton and Edgar, 1998; Chell and Brand, 2010; Cheng et al., 2011; Sousa-Nunes et al., 2011). This late larval brain sparing requires the activity of Anaplastic Lymphoma Kinase (Alk), which bypasses the growth requirements for InR and Tor (Cheng et al., 2011).

In contrast to the well-described NBs in the central brain and ventral nerve cord that form in the embryo, their counterparts in the visual system form much later, during larval stages when body growth depends strictly upon dietary nutrients. These visual system NBs generate neurons and glia that make up the optic lobe (OL) of the *Drosophila* CNS, which integrates the visual input from innervating photoreceptors of the adult retina. The medulla region of the OL has recently emerged as a model for mammalian brain development as it develops via an early

symmetric NE expansion phase followed by conversion of NE into NBs that divide asymmetrically (Egger et al., 2007; Hofbauer and Camposortega, 1990). Upon asymmetric divisions, medulla NBs sequentially express a series of temporal transcription factors that determine the identity of the progeny depending on their birth order (Hasegawa et al., 2011; Maurange, 2012; Morante et al., 2011; X. Li, T. Erclik, C. Bertet, and C. Desplan, personal communication). The switch from proliferative symmetric to neurogenic asymmetric divisions in the OL is controlled by a proneural wave that sweeps through the NE in a medial-to-lateral direction and triggers the conversion to medulla NBs. Elegant studies have demonstrated that progression of the proneural wave is promoted by epidermal growth factor receptor signaling from the medial edge of the NE and counteracted by the NOTCH, JAK/STAT, and FAT/HIPPO signals (Egger et al., 2010; Reddy et al., 2010; Wang et al., 2011; Yasugi et al., 2008, 2010). However, the underlying mechanisms that coordinate these patterning signals to balance symmetric and asymmetric divisions during the course of development are not yet clear.

Here, we investigate how dietary nutrients impact upon the development of the *Drosophila* OL. By using dietary, hormonal, and genetic manipulations, we find that InR/TOR signaling promotes the early symmetric expansion, whereas ecdysone schedules the late asymmetric neurogenic phase. We also find that these two temporally distinct phases exhibit differential nutrient sensitivities, which form the mechanistic basis of an adaptive starvation response preserving neuronal diversity at the expense of OL growth and neuronal number.

RESULTS

The Medulla Switches from Nutrient-Sensitive to -Insensitive Phases of Proliferation

To investigate the impact of nutrients on the OL, we first analyzed a developmental time course under optimal diet. Immunostaining revealed three main phases of medulla development (Figure 1A). During phase 0 (0–12 hr after larval hatching [ALH]), NE cells are small and do not divide (Figure 1B). Phase 1 (12–60 hr ALH) is characterized by symmetric divisions, leading to a large expansion of the NE (Figure 1C). During phase 2 (60–120 hr ALH), NE-to-NB conversion is stimulated and the NE regresses, presumably as a result of proneural wave progression combined with decreased proliferation (Figures 1D and S1). From 120 hr (24 hr after puparium formation [APF]) onward, NE and NBs can no longer be identified, indicating that neurogenesis has terminated (Figure 1E). In summary, during medulla development, the NE transitions from expansion (phase 1) to

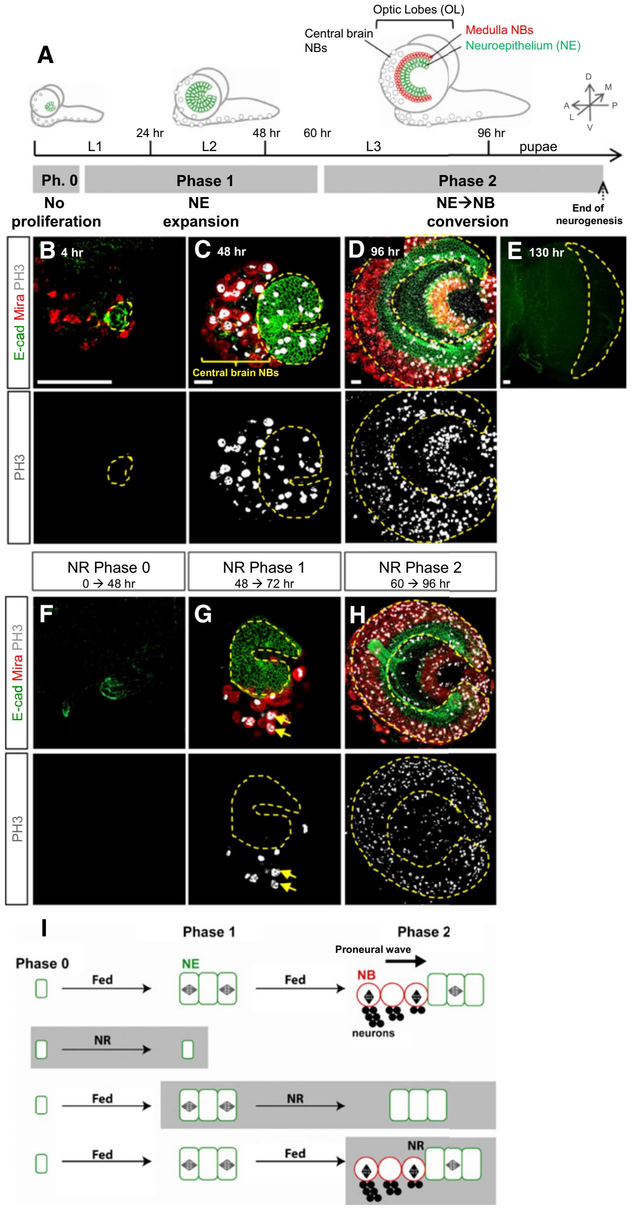

Figure 1. Nutritional Signals Control the Three Phases of NE Development

In all figures of the article, images are projections of several confocal sections (except if stated otherwise).

(A) Schematic drawings representing lateral views of a larval CNS from early L1 to late L3. Central brain and nerve cord neuroblasts (NBs) are represented as gray circles. In the OL, medulla NBs are represented as red circles, and neuroepithelial cells (NEs) are represented as green rectangles. Medulla NBs are smaller and also more densely packed than their central brain counterparts and form a characteristic horseshoe-shaped strip adjacent to the medial edge of the NE. 3D axis are presented as A-P, anterior-posterior; D-V, dorsal-ventral; M-L, medial-lateral.

(B–E) Pictures show larval OL during the three periods of medulla development and a frontal view of a pupal OL (130 hr). The NE is stained by E-Cadherin (E-Cad; green), NBs are marked with Mira (red), and mitotic cells are marked with PH3 (white). The dotted yellow line delineates the medulla (NE and NBs). (B) Phase 0: inactive NE. (C) Phase 1: expansion. (D) Phase 2: NE → NB conversion. (E) At midpupae, no more NE cells and NBs are detected.

(F) The NE of larvae submitted to NR conditions for 48 hr from hatching (phase 0) never initiate proliferation.

(G) The NE of larvae starved from 48 hr ALH (phase 1) to 96 hr arrests proliferation (no PH3^{+} NE cells) and does not undergo NB conversion. Yellow arrows indicate central brain NBs that are still dividing in these conditions.

(H) NR during phase 2 (from 60 to 96 hr) impacts neither on neural proliferation nor on NE → NB conversion.

(I) Schematic representation of the results obtained from the NR experiments during the three periods of medulla development (F–H).

See also Figure S1.

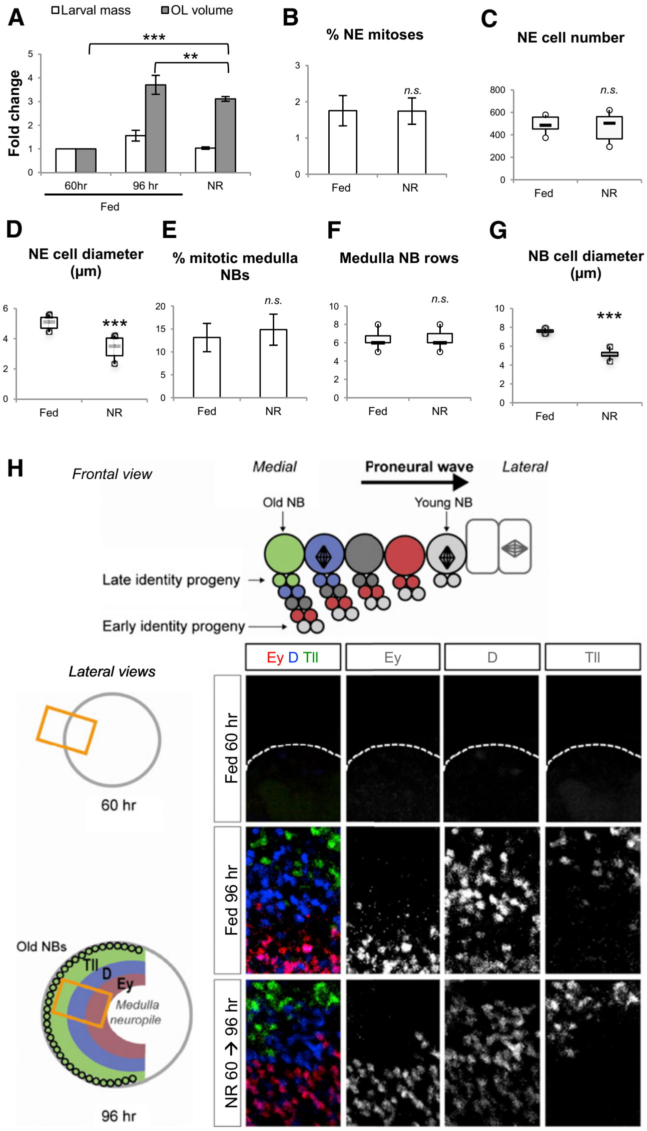

Figure 2. During Phase 2, Neural Progenitor Division and Neuronal Diversity Are Protected from NR

(A) Larval body mass stops increasing under 60 → 96 hr NR conditions. In contrast, the optic lobe continues growing reaching 84% of its normal volume. Fold changes have been calculated from the following measurements: Larval mass: 60 hr fed (m = 1.45 mg, n = 30, SD = 0.09); 96 hr fed (m = 1.92 mg, n = 30, SD = 0.04); 96 hr NR (m = 1.475, n = 54, SD = 0.03); $p < 0.001$. OL diameters: fed 60 hr (m = 148.5 µm, n = 6, SD = 16.8); 96 hr fed (m = 230.6 µm, n = 24, SD = 26.2); 96 hr NR (m = 217.5 µm, n = 20, SD = 16.5).

(B) The mitotic index in the NE does not significantly differ after 60 → 96 hr NR compared to fed larvae. Fed (m = 1.75, n = 8 OL, SD = 0.4); NR (m = 1.74, n = 6 OL, SD = 0.4); $p > 0.05$.

(C) After 60 → 96 hr NR, the total number of NE cells in the medulla does not significantly differ compared to fed larvae. Fed (m = 493, n = 8, SD = 71); NR (m = 471, n = 6, SD = 133); $p > 0.05$.

(D) After 60 → 96 hr NR, the apical diameter of NE cells significantly decreases compared to fed conditions. Fed (n = 6 OL, m = 5.00, SD = 0.46), NR (n = 6 OL, m = 3.40, SD = 0.76). ***$p < 0.001$.

(E) After 60 → 96 hr NR, the percentage of $PH3^+$ medulla NBs does not significantly differ compared to fed larvae. Fed (m = 13.1%, n = 5 OL, SD = 3.0); NR (m = 14.8%, n = 4 OL, SD = 3.0); $p > 0.05$.

(F) After 60 → 96 hr NR, the width of the NB strip does not significantly differ compared to fed larvae. Fed (m = 6.2, n = 14, SD = 0.8); NR (m = 6.3, n = 16, SD = 0.8); $p > 0.05$.

(G) After 60 → 96 hr NR, the diameter of medulla NBs significantly decreases compared to fed conditions. Fed (n = 5 OL, m = 7.6, SD = 0.2), NR (n = 4 OL, m = 5.1, SD = 0.6). ***$p < 0.001$.

(H) A frontal cross-section view of the OL showing the proneural wave traversing the NE in a medial to lateral direction. Medulla NBs are represented as large circles, progeny as small circles and NE cells are represented as rectangles. Converted NBs express different temporal factors endowing progeny with different identity (color code) (X. Li, T. Erclik, C. Bertet, and C. Desplan, personal communication). On the lateral cross-section through 96 hr medulla, concentric layers of Ey^+, D^+, and Tll^+ cells are visible (respectively colored in red, blue, and green on the scheme) representative of their birth order. At 60 hr, medulla neurons have not been generated yet. The curved edge of the optic lobe is indicated by a white dotted line. Between 60 and 96 hr, early (Ey^+) and late (D^+ and Tll^+) identity progeny are generated in the medulla of both fed and NR larvae. Ey, red; D, blue; Tll, green.

regression (phase 2) and is progressively converted into neurogenic NBs. We then investigated whether phases 0–2 are sensitive to severe nutrient restriction (NR), by challenging larvae with an amino-acid-free diet. Consistent with a previous study (Britton and Edgar, 1998), larvae subject to NR from hatching (phase 0) retain a small NE with no mitotic cells, showing that dietary amino acids are required to activate growth and proliferation (Figure 1F). If NR is applied from 48 (phase 1) to 96 hr, no mitoses are visible in the NE, but dividing central brain NBs are nevertheless observed (Figure 1G). Under these NR conditions, only very few medulla NBs are produced from the NE. Thus, the continuous presence of dietary nutrients is necessary to sustain mitotic activity during NE expansion and also to promote the NE-to-NB conversion. When larvae are transferred to NR just after 60 hr (phase 2), the strip of medulla NBs at 96 hr is about as wide as in fed controls, with both NE cells and NBs continuing to divide (Figure 1H). This indicates that, once phase 2 has been initiated, both proneural wave progression and cell division remain largely unaffected by NR. Together, these experiments suggest that there are two phases during medulla development that are differentially sensitive to dietary nutrients: an early phase that requires dietary nutrients to activate and sustain NE expansion, and a later phase that can sustain neural proliferation and convert NE cells to NBs without dietary inputs (Figure 1I).

Medulla Progenitor Numbers and Neuronal Diversity Are Protected during Phase 2 NR

To determine whether growth, as well as proliferation, is protected from withdrawal of dietary amino acids during phase 2, we conducted a quantitative analysis of the OL. NR from 60 to 96 hr does not significantly change larval body mass but the OL volume increases 3-fold, reaching ~84% of its normal volume (Figure 2A). NR did not significantly reduce cell number or the mitotic index of the NE and medulla NBs, but it did significantly decrease cell size (Figures 2B–2G). Thus, progenitor proliferation and, to a lesser extent, growth during phase 2 are protected from NR, irrespective of whether the mode of division is symmetric or asymmetric. We then tested whether NR during phase 2 affects the ability of medulla NBs to generate their normal temporal repertoire of neurons. As new medulla NBs are being converted, they start to sequentially express a series

Cell PRESS
Open ACCESS

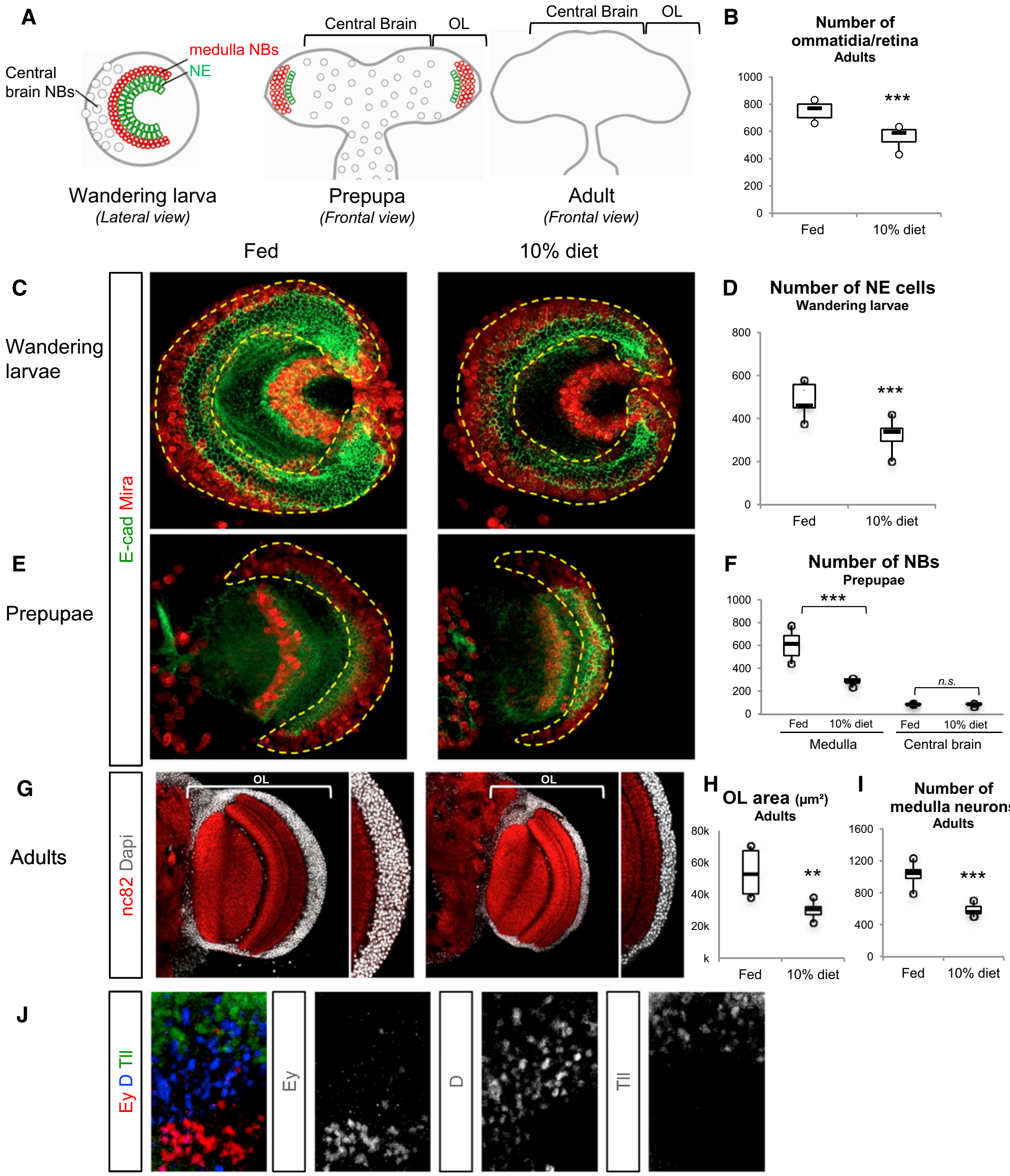

Figure 3. Reduction of the Neural Progenitor Pool Size in Response to Suboptimal Nutritional Conditions Leads to Fewer Neurons Being Generated

(A) Schematic drawings of the *Drosophila* OL in the CNS from three postembryonic stages. In the adult brain, neurogenesis has terminated and NE cells and NBs are not detected.

(B) The number of ommatidia that compose the retina significantly decreases in adults that have developed in the 10% diet compared to fed condition. Fed (m = 754, n = 11, SD = 59); 10% (m = 568, n = 14, SD = 63); ***p < 0.001.

(C) Lateral views of OL from wandering larva reared in fed or 10% diet conditions.

(D) The number of NE cells in wandering larvae significantly decreases in the 10% diet condition compared to fed. Fed (m = 516, n = 10 SD = 127); 10% (m = 354, n = 7 SD = 77); ***p < 0.001.

(E) Frontal view of a hemibrain from a 12-hr-old pupae.

(C and E) The dotted yellow line delineates the medulla (NE and NBs). E-cad, green; Mira, red.

(F) The number of NBs is significantly reduced in the medulla of 12-hr-old pupae reared 10% diet condition compared to fed. In contrast, it does not differ in the central brain (CB). Medulla: fed (m = 604, n = 6, SD = 126), 10% (m = 239, n = 5, SD = 32); ***p < 0.001; CB: fed (m = 85, n = 5, SD = 6), 10% (m = 83, n = 5 SD = 12); p > 0.05.

(legend continued on next page)

of transcription factors including Ey, D, and Tll (X. Li, T. Erclik, C. Bertet, and C. Desplan, personal communication). Consequently, medulla NBs successively generate Ey$^+$, D$^+$, and Tll$^+$ progeny (neurons and glia) in concentric layers within the OL medulla, the order of which reflects their birth order (Figure 2H) (Hasegawa et al., 2011; Morante et al., 2011; X. Li, T. Erclik, C. Bertet, and C. Desplan, personal communication). There are no Ey$^+$, D$^+$, or Tll$^+$ cells present in the anterior half of the medulla region at 60 hr, but all three types of progeny are appropriately generated in the medulla of wandering larvae subjected to NR from 60 to 96 hr (Figure 2H). Together, these results suggest that NE and medulla NB proliferation is protected against NR during phase 2, thus allowing medulla NBs to generate their temporal repertoire of neural progeny.

Nutrients Promote NE Expansion during Phase 1 via the TOR/InR/PI3K Network

The NR experiments thus far reveal that NE proliferation during phase 1 is highly sensitive to dietary nutrients. We therefore tested the TOR/InR pathway, as this is known to sense environmental nutrients and to control cell growth (Figure S2A). The NE of homozygous $Tor^{\Delta P}$ and $Akt1^{1}$ mutant larvae (Zhang et al., 2000) does not expand, even up to 48 hr ALH (Figure S2B). In addition, we find that phosphorylated 4E-BP (a readout for Tor activity) is highly expressed in the expanding NE of fed larvae during phase 1 but becomes undetectable after 24 hr NR, correlating with proliferation arrest (Figure S2C). Together, these results suggest that larval feeding activates the TOR/InR network and thus promotes NE expansion.

Medulla Neuronal Diversity Is Protected at the Expense of Neuronal Numbers during Sustained Dietary Restriction

To investigate the impact of a sustained nutritional challenge spanning all phases of medulla development (Figure 3A), we reared animals throughout larval life on standard food diluted ten times (10% diet). This diet permits completion of development, albeit delayed by 2–4 days, and gives rise to smaller-than-normal adult flies (Layalle et al., 2008). We now show that the eyes of such flies possess 25% less ommatidia than fed controls (Figure 3B). In late 10% diet larvae, we find a decrease in the number of NE cells (Figures 3C and 3D) that correlates, in prepupae (12 hr APF), with reduced medulla NB numbers compared to fed controls (Figures 3E and 3F). In contrast, and consistent with a previous NR study (Cheng et al., 2011), the 10% diet did not significantly reduce the number of central brain NBs (Figure 3F). Nevertheless, sustained dietary restriction appears to reduce NE cell number in a way consistent with the nutrient sensitivity of phase 1, leading to a smaller pool of medulla progenitors. We next assessed the effects of the 10% diet upon the adult brain and observed a drastic reduction in the area of the OL and in the number medulla neurons (up to 40% per section) (Figures 3G–3I). Thus, in response to larval undernutrition, the neural progenitor pool is reduced, leading to fewer neurons being generated in adults. However, larvae subjected to the dietary restriction remain able to generate concentric layers of Ey$^+$, D$^+$, and Tll$^+$ medulla progeny, showing that neuronal temporal diversity is preserved during the neurogenic phase (Figure 3J).

Ecdysone Signaling Represses Dl in the NE, Limits the Progenitor Pool, and Triggers Neurogenesis

We then sought to identify the NR-resistant signal that initiates neurogenesis during phase 2. The end of phase 1 correlates with the first of a series of three L3 ecdysone bursts from the prothoracic gland (Mirth and Shingleton, 2012; Warren et al., 2006). Thus, ecdysone could be responsible for promoting the phase-1-to-phase-2 transition. We find that the common isoform of ecdysone receptor (EcR) is expressed in NE cells of late larvae and that an *EcRE-lacZ* transgenic reporter of ecdysone signaling (Brennan et al., 1998; Schwedes et al., 2011) is activated in the NE of late L3 larvae (Figures S3A and 4A). Thus, the onset of EcR signaling in the NE temporally correlates with the major period of NE-to-NB conversion. To investigate further the relationship between ecdysone and NE-to-NB conversion, we first performed ex vivo experiments. CNSs from early L3 larvae (phase 1) explanted to a high concentration of ecdysone undergo precocious NE depletion, thus limiting the final number of medulla NBs that are generated (Figure S3B). Moreover, the NE of *molting defective* (mld^{DTS3}) mutant larvae, in which the ecdysone pulses are abrogated (Holden et al., 1986), continues expanding for several days while NE-to-NB conversion is reduced (Figure S3C). Together, these results demonstrate that EcR signaling during L3 is both necessary and sufficient to stimulate the NE-to-NB conversion with a concomitant reduction in the pool of NE cells. We then investigated if the requirement for EcR signaling was autonomous to NE cells. An efficient way to suppress both the activation and derepression functions of the ecdysone response is to express a dominant-negative EcR (Brown et al., 2006; Mirth et al., 2009). Control and EcR^{DN}-expressing clones were induced in the medulla NE during early larval stages (24 hr) and examined at 96 hr. Control clones span the NE and NB regions, separated by a sharp and linear E-Cadherin (E-cad)/Mira boundary (Figure 4B). In contrast, EcR^{DN} clones display a medially displaced boundary (Figure 4B), a phenotype that has been attributed to a delayed proneural wave (Reddy et al., 2010; Yasugi et al., 2008, 2010). This interpretation is supported by the increase in NE cells and the reduction in neurons observed in EcR^{DN} clones relative to control clones (Figure S3D). We then sought to identify the downstream

(G) Single frontal confocal section of a hemibrain from a 1-day-old adult. DAPI (gray) stains nuclei and nc82 (red) stains neuropils.

(H) The area of the medulla is significantly reduced in 10% diet compared to fed animals. Fed (m = 53724, n = 8, SD = 14,636); 10% (m = 30,240, n = 5, SD = 5,459); **$p < 0.01$.

(I) The number of medulla neurons per confocal section significantly decreases in 10% compared to fed condition. Fed (m = 1,070, n = 8, SD = 130); 10% (m = 607, n = 5, SD = 80); ***$p < 0.001$.

(J) As in the Fed condition, early (Ey$^+$) and late (D$^+$ and Tll$^+$) identity neurons are generated in the medulla of wandering larvae reared in the 10% diet. Ey, red; D, blue; Tll, green.

Cell PRESS
Open ACCESS

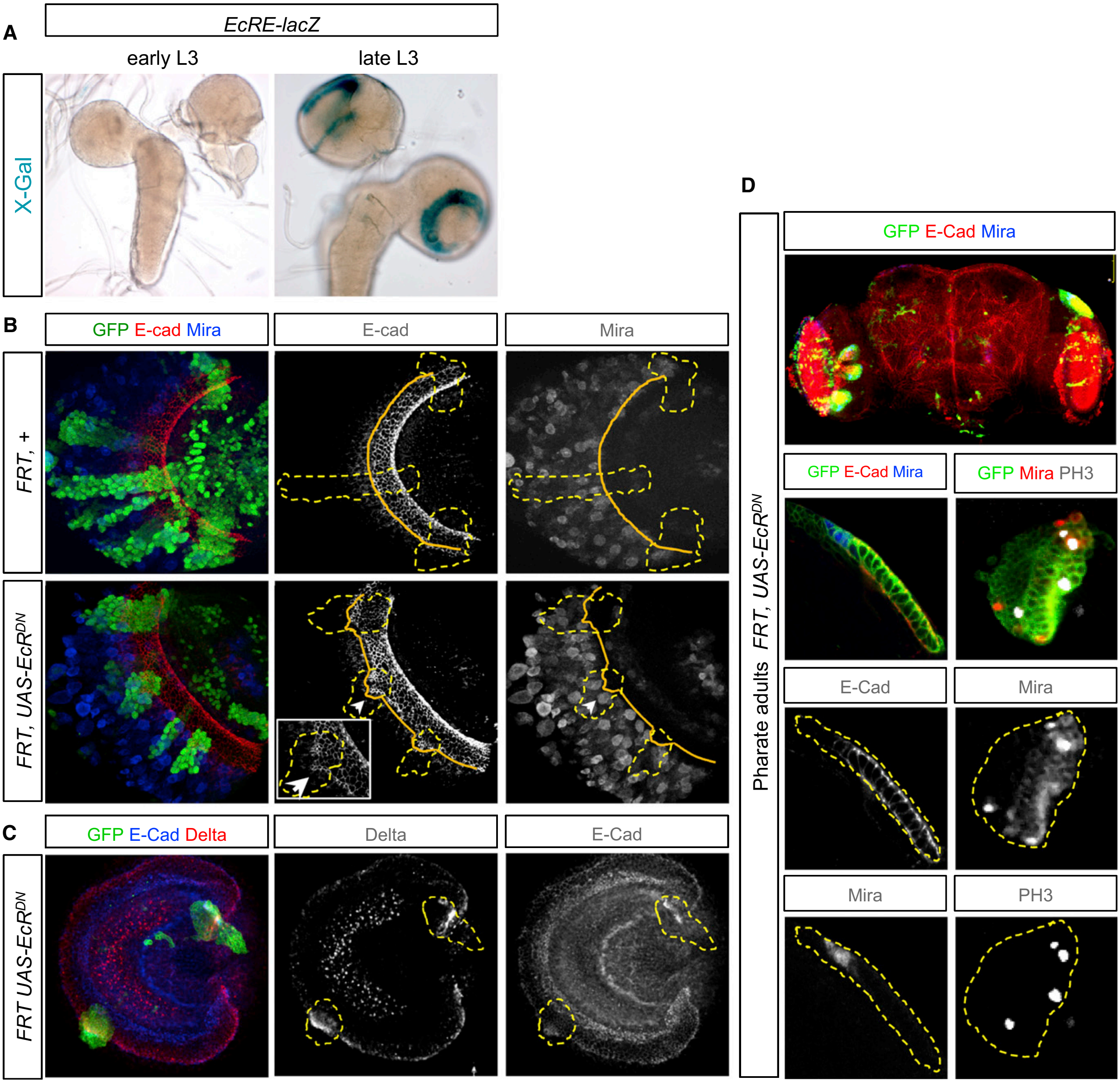

Figure 4. Ecdysone Triggers NE → NB Conversion through the Downregulation of Delta and Is Required Cell Autonomously to Complete NE Elimination

(A) X-gal staining demonstrates that *EcRE-lacZ* is specifically activated in the NE of late L3, but not in early L3.

(B) In late L3, wild-type MARCM clones span the NE (E-cad, red) and NB (Mira, blue) populations. Clones misexpressing EcR^{DN} exhibit a delayed proneural wave, as shown by the systematic presence of more medial E-cad staining inside clones compared to surrounding tissue.

(C) Delta (red) is upregulated in EcR^{DN} clones throughout the NE (blue).

(D) EcR^{DN} GFP^{+} clones in the pharate adult retain NE cells and NBs (E-cad, red; Mira, blue; see higher magnifications) that are still proliferating (Mira, red; PH3, white).

See also Figure S3.

targets of EcR signaling relevant to the NE-to-NB conversion in the medulla. The Notch pathway has been shown to regulate the NE-to-NB conversion (Egger et al., 2010; Wang et al., 2011; Yasugi et al., 2010). We detect strong expression of the Notch ligand, Delta, in the NE during phase 1, with particularly high levels at or close to the NE/NB boundary. Interestingly, during phase 2, Delta in the NE becomes strongly downregulated (Figure S3E). In EcR^{DN} clones, however, there is a striking

failure to downregulate Delta, which is most pronounced at the NE/NB boundary (Figure 4C). Moreover, overexpression of Delta in medulla clones is sufficient to shift the NE/NB border more medially, thus phenocopying EcR^{DN} expression (Figure S3F), whereas loss of Delta in EcR^{DN} clones abrogates the delay (Figure S3G). Strikingly, in EcR^{DN} clones, the NE remains present in pharate adults, continuing to proliferate and to generate NBs and neurons long after neurogenesis in the surrounding wild-type tissue has terminated (Figure 4D). Thus, EcR signaling is required in the NE for the timely termination of neurogenesis. In summary, these experiments demonstrate that ecdysone induces the symmetric-to-asymmetric switch through the repression of the Delta/Notch pathway in the NE. This late developmental event limits the neural progenitor pool and schedules the neurogenic phase to the diet-insensitive period.

DISCUSSION

In addition to governing organismal size (Colombani et al., 2005; Layalle et al., 2008), we have shown that Ilps and ecdysone also determine the size of the NSC pool in the *Drosophila* visual system. During early larval stages, nutrients signal via the InR/TOR pathway to promote NE cell growth and symmetric divisions. This nutrient response, combined with high levels of Notch signaling, triggers a rapid expansion of the NE. During L3, ecdysone downregulates Delta in the NE, thus accelerating progression of the proneural wave that regulates the balance between proliferation and neurogenesis. This leads to the termination of NE expansion and the progressive conversion of all NE cells to medulla NBs. Thus, Ilps and ecdysone exert antagonistic actions on the NE, respectively, promoting and terminating its expansion (Figure S4). This system allows the mode of stem cell division in the medulla to be coordinated with the growth of the organism. Given the evolutionary conservation of the InR/TOR network and of some nuclear receptors (King-Jones and Thummel, 2005), the mechanisms underlying the regulation of the NE-to-radial glia switch might also be systemic in mammals and linked to Notch signaling. We have also shown that an early nutrient sensitivity of NE proliferation leads to a reduced NSC pool in poorly fed larvae, and to a reduced number of neurons in the adult medulla. This phenomenon demonstrates that the NSC pool size is diet dependent in the OL, in contrast to other regions of the CNS. This may reflect the need to coordinate the numbers of cells in the OL with those of the incoming photoreceptors from the eye disc ommatidia, which we find are also reduced by food restriction. This matching system may thus facilitate one-to-one retinotopic mapping in the adult. We also note that medulla progenitors are able to maintain near normal numbers of cell divisions during NR in phase 2, even though they exhibit a significant growth reduction. Thus, the Alk-dependent growth protection mechanisms operating in NBs of the central brain and nerve cord (Cheng et al., 2011) are unlikely to apply to the same extent to the NBs of the visual system. Importantly, regulation of the NSC pool size during the early diet-sensitive phase 1, combined with protection of ecdysone-mediated neurogenesis during the late diet-insensitive period, permits a reduction of adult neuronal number without loss of neuronal diversity (Figure S4). Together with work on Alk (Cheng et al., 2011), our study reveals the existence of region specific mechanisms for brain sparing and suggests possible cellular and molecular routes by which early nutrient restriction may affect mammalian brain development and growth.

EXPERIMENTAL PROCEDURES

Larval Dietary Manipulations

Drosophila were raised at 25°C on standard medium (8% cornmeal/8% yeast/1% agar) unless indicated otherwise. For nutrient restriction experiments, hatching larvae were transferred to 20% sucrose in PBS; phases 1 and 2 larvae were transferred on 1% agar/PBS medium. For 10% diet experiments, fly food was obtained by diluting ten times the conventional food.

Image Processing and Statistical Tests

Confocal images were acquired on a Leica SP5, Zeiss lsm510, and Zeiss lsm 780.

For further details, please refer to Extended Experimental Procedures.

SUPPLEMENTAL INFORMATION

Supplemental Information includes Extended Experimental Procedures and four figures and can be found with this article online at http://dx.doi.org/10.1016/j.celrep.2013.02.006.

LICENSING INFORMATION

This is an open-access article distributed under the terms of the Creative Commons Attribution License, which permits unrestricted use, distribution, and reproduction in any medium, provided the original author and source are credited.

ACKNOWLEDGMENTS

We are grateful to P.L. Bardet, B. Bello, H. Jasper, P. Leopold, R. Palmer, J. Skeath, and H. Stocker for flies and antibodies. We also acknowledge the Bloomington and Vienna Drosophila RNAi centers for fly stocks, and the DSHB for monoclonal antibodies, and the C.M. group for continuous discussions. We thank R. Kelly, P. Durbec, V. Bertrand, and L. Cheng for critical reading of the manuscript, and C. Desplan for sharing unpublished data. E.L. is supported by the Association pour la Recherche sur le Cancer, A.P.G. by the Medical Research Council (U117584237), and C.M. by the ATIP-CNRS program and the Fondation pour la Recherche Médicale.

Received: July 2, 2012
Revised: January 22, 2013
Accepted: February 5, 2013
Published: March 7, 2013

REFERENCES

Aguirre, A., Rubio, M.E., and Gallo, V. (2010). Notch and EGFR pathway interaction regulates neural stem cell number and self-renewal. Nature *467*, 323–327.

Brennan, C.A., Ashburner, M., and Moses, K. (1998). Ecdysone pathway is required for furrow progression in the developing Drosophila eye. Development *125*, 2653–2664.

Britton, J.S., and Edgar, B.A. (1998). Environmental control of the cell cycle in Drosophila: nutrition activates mitotic and endoreplicative cells by distinct mechanisms. Development *125*, 2149–2158.

Brogiolo, W., Stocker, H., Ikeya, T., Rintelen, F., Fernandez, R., and Hafen, E. (2001). An evolutionarily conserved function of the Drosophila insulin receptor and insulin-like peptides in growth control. Curr. Biol. *11*, 213–221.

Brown, H.L., Cherbas, L., Cherbas, P., and Truman, J.W. (2006). Use of time-lapse imaging and dominant negative receptors to dissect the steroid receptor control of neuronal remodeling in Drosophila. Development *133*, 275–285.

Chell, J.M., and Brand, A.H. (2010). Nutrition-responsive glia control exit of neural stem cells from quiescence. Cell *143*, 1161–1173.

Cheng, L.Y., Bailey, A.P., Leevers, S.J., Ragan, T.J., Driscoll, P.C., and Gould, A.P. (2011). Anaplastic lymphoma kinase spares organ growth during nutrient restriction in Drosophila. Cell *146*, 435–447.

Colombani, J., Bianchini, L., Layalle, S., Pondeville, E., Dauphin-Villemant, C., Antoniewski, C., Carré, C., Noselli, S., and Léopold, P. (2005). Antagonistic actions of ecdysone and insulins determine final size in Drosophila. Science *310*, 667–670.

Egger, B., Boone, J.Q., Stevens, N.R., Brand, A.H., and Doe, C.Q. (2007). Regulation of spindle orientation and neural stem cell fate in the Drosophila optic lobe. Neural Dev. *2*, 1.

Egger, B., Gold, K.S., and Brand, A.H. (2010). Notch regulates the switch from symmetric to asymmetric neural stem cell division in the Drosophila optic lobe. Development *137*, 2981–2987.

Falk, S., Wurdak, H., Ittner, L.M., Ille, F., Sumara, G., Schmid, M.T., Draganova, K., Lang, K.S., Paratore, C., Leveen, P., et al. (2008). Brain area-specific effect of TGF-beta signaling on Wnt-dependent neural stem cell expansion. Cell Stem Cell *2*, 472–483.

Farkas, L.M., and Huttner, W.B. (2008). The cell biology of neural stem and progenitor cells and its significance for their proliferation versus differentiation during mammalian brain development. Curr. Opin. Cell Biol. *20*, 707–715.

Götz, M., and Huttner, W.B. (2005). The cell biology of neurogenesis. Nat. Rev. Mol. Cell Biol. *6*, 777–788.

Gruenwald, P. (1963). Chronic fetal distress and placental insufficiency. Biol. Neonat. *5*, 215–265.

Hasegawa, E., Kitada, Y., Kaido, M., Takayama, R., Awasaki, T., Tabata, T., and Sato, M. (2011). Concentric zones, cell migration and neuronal circuits in the Drosophila visual center. Development *138*, 983–993.

Hofbauer, A., and Camposortega, J.A. (1990). Proliferation pattern and early differentiation of the optic lobes in Drosophila-melanogaster. Rouxs Arch. Dev. Biol. *198*, 264–274.

Holden, J.J.A., Walker, V.K., Maroy, P., Watson, K.L., White, B.N., and Gausz, J. (1986). Analysis of molting and metamorphosis in the ecdysteroid-deficient mutant L(3)3dts of Drosophila-melanogaster. Dev. Genet. *6*, 153–162.

Ikeya, T., Galic, M., Belawat, P., Nairz, K., and Hafen, E. (2002). Nutrient-dependent expression of insulin-like peptides from neuroendocrine cells in the CNS contributes to growth regulation in Drosophila. Curr. Biol. *12*, 1293–1300.

Jacob, J., Maurange, C., and Gould, A.P. (2008). Temporal control of neuronal diversity: common regulatory principles in insects and vertebrates? Development *135*, 3481–3489.

King-Jones, K., and Thummel, C.S. (2005). Nuclear receptors—a perspective from Drosophila. Nat. Rev. Genet. *6*, 311–323.

Layalle, S., Arquier, N., and Léopold, P. (2008). The TOR pathway couples nutrition and developmental timing in Drosophila. Dev. Cell *15*, 568–577.

Maurange, C. (2012). Temporal specification of neural stem cells: insights from Drosophila neuroblasts. Curr. Top. Dev. Biol. *98*, 199–228.

Mirth, C.K., and Shingleton, A.W. (2012). Integrating body and organ size in Drosophila: recent advances and outstanding problems. Front Endocrinol (Lausanne) *3*, 49.

Mirth, C.K., Truman, J.W., and Riddiford, L.M. (2009). The ecdysone receptor controls the post-critical weight switch to nutrition-independent differentiation in Drosophila wing imaginal discs. Development *136*, 2345–2353.

Morante, J., Erclik, T., and Desplan, C. (2011). Cell migration in Drosophila optic lobe neurons is controlled by eyeless/Pax6. Development *138*, 687–693.

Okano, H., and Temple, S. (2009). Cell types to order: temporal specification of CNS stem cells. Curr. Opin. Neurobiol. *19*, 112–119.

Randhawa, R., and Cohen, P. (2005). The role of the insulin-like growth factor system in prenatal growth. Mol. Genet. Metab. *86*, 84–90.

Reddy, B.V., Rauskolb, C., and Irvine, K.D. (2010). Influence of fat-hippo and notch signaling on the proliferation and differentiation of Drosophila optic neuroepithelia. Development *137*, 2397–2408.

Sahara, S., and O'Leary, D.D. (2009). Fgf10 regulates transition period of cortical stem cell differentiation to radial glia controlling generation of neurons and basal progenitors. Neuron *63*, 48–62.

Schwedes, C., Tulsiani, S., and Carney, G.E. (2011). Ecdysone receptor expression and activity in adult Drosophila melanogaster. J. Insect Physiol. *57*, 899–907.

Skeath, J.B., and Thor, S. (2003). Genetic control of Drosophila nerve cord development. Curr. Opin. Neurobiol. *13*, 8–15.

Sousa-Nunes, R., Yee, L.L., and Gould, A.P. (2011). Fat cells reactivate quiescent neuroblasts via TOR and glial insulin relays in Drosophila. Nature *471*, 508–512.

Wang, W., Liu, W., Wang, Y., Zhou, L., Tang, X., and Luo, H. (2011). Notch signaling regulates neuroepithelial stem cell maintenance and neuroblast formation in Drosophila optic lobe development. Dev. Biol. *350*, 414–428.

Warren, J.T., Yerushalmi, Y., Shimell, M.J., O'Connor, M.B., Restifo, L.L., and Gilbert, L.I. (2006). Discrete pulses of molting hormone, 20-hydroxyecdysone, during late larval development of Drosophila melanogaster: correlations with changes in gene activity. Dev. Dyn. *235*, 315–326.

Yasugi, T., Umetsu, D., Murakami, S., Sato, M., and Tabata, T. (2008). Drosophila optic lobe neuroblasts triggered by a wave of proneural gene expression that is negatively regulated by JAK/STAT. Development *135*, 1471–1480.

Yasugi, T., Sugie, A., Umetsu, D., and Tabata, T. (2010). Coordinated sequential action of EGFR and Notch signaling pathways regulates proneural wave progression in the Drosophila optic lobe. Development *137*, 3193–3203.

Zhang, H., Stallock, J.P., Ng, J.C., Reinhard, C., and Neufeld, T.P. (2000). Regulation of cellular growth by the Drosophila target of rapamycin dTOR. Genes Dev. *14*, 2712–2724.

Supplemental Information

EXTENDED EXPERIMENTAL PROCEDURES

Fly Lines

For generating MARCM clones (Lee and Luo, 1999), the following stocks were used. For the X chromosome: *w hsFLP1, FRT19A, tubP-GAL80LL1; UAS-nlsLacZ20b, UAS-mCD8::GFPLL5; tub-GAL4LL7*. For the III chromosome: *w, tub-GAL4, UAS-nlsGFP::6xmyc::NLS, hsFLP122; FRT82B, tubP-GAL80 LL3/TM6B*. Flip-out clones were generated using *hs-FLP; Act5C < CD2 > GAL4*, *UAS-GFP*. *FRT82B DlrevF10, e/ TM3* (from F. Schweisguth) ; *FRT82B, Akt1*[1] (Staveley et al., 1998). *UAS-EcR.B1-DeltaC655.F645A; UAS-EcR.B1-DeltaC655.W650A; EcRE-lacZ, Oregon; UAS-Delta; tub-GAL80*^{ts}*; DTS3 ; Tor*$^{\Delta P}$, FRT40A stocks were provided by the Bloomington Stock Center. Both EcR dominant-negative versions (EcRB1F645A and EcRB1W650A) gave similar results. Images shown are for EcRB1^{F645A} (called EcR^{DN} in the study).

In Vitro CNS Culture

In vitro culture was performed based on (Awad and Truman, 1997). Larvae were surface sterilized in a solution of 70% ethanol for 5-7 min, rinsed in sterile water, and dissected in Schneider's insect cell culture medium (GIBCO) using sterile tools. Tissues were transferred on a drop of culture medium with 10% fetal bovine serum and 1% of an antibiotic/antimycotic solution containing 10,000 units/mL penicillin, 10 mg/mL streptomycin, 25 μg/mL insulin (Sigma), 1 μg/mL to 1mg/mL 20-H-ecdysone (Sigma), and 50 μL/mL of fly extract prepared as in (Currie et al., 1988). Cultures were incubated at 25°C in humidity chambers. Cultured tissues were fixed and stained as described below.

Immunohistochemistry

Optic lobes were dissected for fixation when control larvae reached the wandering stage that precedes pupariation. For pupal staging, wandering larvae of NR and fed condition were left overnight and early P5 pupae (aged about 12 hr) were collected based on morphological criteria (Bainbridge and Bownes, 1981). Tissues were fixed from 5 to 15 min in 4% formaldehyde/PBS depending on the primary antibody. Stainings were performed in 0.4% triton/PBS with antibody incubations separated by several washes. Tissues were then transferred in Vectashield for image acquisition. Primary antibodies were: chicken anti-GFP (1:1000, Tebubio), mouse anti-nc82 (1:20, DSHB), mouse anti-Mira (1:50), mouse anti-Delta (1:200, DSHB), rat anti-ECad (1:50, DSHB), rabbit anti-PH3 (1:500, Millipore), rat anti-PH3 (1:500, Abcam), rabbit anti-Phospho-4E-BP (1:75, Cell Signaling Technology), rabbit anti-Tll (1:100, J. Reinitz), mouse anti-Ey (1:30, DHSB), guinea-pig anti-D (1:50). Adequate combinations of secondary antibodies (Jackson ImmunoResearch) were used to reveal expression patterns.

Image Processing and Statistical Tests

Volocity, ImageJ and Zen were used to process confocal data. Figure 2: OL Volumes are extrapolated from diameters measured from confocal sections traversing the center of the lobes. Apical diameters of NE cells were measured from the average of two orthogonal diameters. Diameters of PH3^{+} medulla NBs were measured from the average of two orthogonal diameters. Figure 3: For an estimation of medulla neuron numbers in the adult, a unique coronal confocal section was taken midway through the posterior half of each fixed adult brain. The total number of medulla neurons per confocal slices in adults reared on normal or 10%-diet conditions was estimated by multiplying the average number of nuclei constituting the thickness of the medulla, with the total number of nuclei along the periphery of the medulla. Nine measurements evenly spaced along the medulla where considered to assess the average thickness. The area is measured from the broadest confocal section. P values were generated using Mann-Whitney U-test for samples $n < 30$. For $n > 30$, P values were calculated assuming equal sample variance, using two-tailed Student's t tests. All quantifications are represented in box and whisker plots and histograms.

SUPPLEMENTAL REFERENCES

Awad, T.A., and Truman, J.W. (1997). Postembryonic development of the midline glia in the CNS of Drosophila: proliferation, programmed cell death, and endocrine regulation. Dev. Biol. *187*, 283–297.

Bainbridge, S.P., and Bownes, M. (1981). Staging the metamorphosis of *Drosophila melanogaster*. J. Embryol. Exp. Morphol. *66*, 57–80.

Currie, D.A., Milner, M.J., and Evans, C.W. (1988). The growth and differentiation in vitro of leg and wing imaginal disc cells from *Drosophila melanogaster*. Development *102*, 805–814.

Lee, T., and Luo, L. (1999). Mosaic analysis with a repressible cell marker for studies of gene function in neuronal morphogenesis. Neuron *22*, 451–461.

Neubueser, D., Warren, J.T., Gilbert, L.I., and Cohen, S.M. (2005). Molting defective is required for ecdysone biosynthesis. Dev. Biol. *280*, 362–372.

Staveley, B.E., Ruel, L., Jin, J., Stambolic, V., Mastronardi, F.G., Heitzler, P., Woodgett, J.R., and Manoukian, A.S. (1998). Genetic analysis of protein kinase B (AKT) in Drosophila. Curr. Biol. *8*, 599–602.

Cell PRESS
Open ACCESS

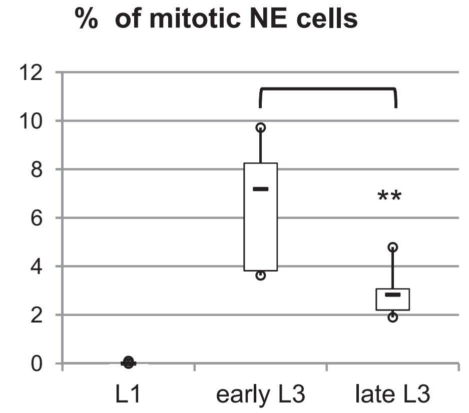

Figure S1. NE Proliferation Rate during Larval Stages, Related to Figure 1

During early L1 stage NE cells are quiescent and do not exhibit PH3 stainings (n = 6, m = 0.01%, SD = 0.04). Early L3 NEs exhibit more mitotic cells (n = 8, m = 6.5%, SD = 2.5) than late L3 NEs (n = 6, mean = 2.9%, SD = 1). **p < 0.01.

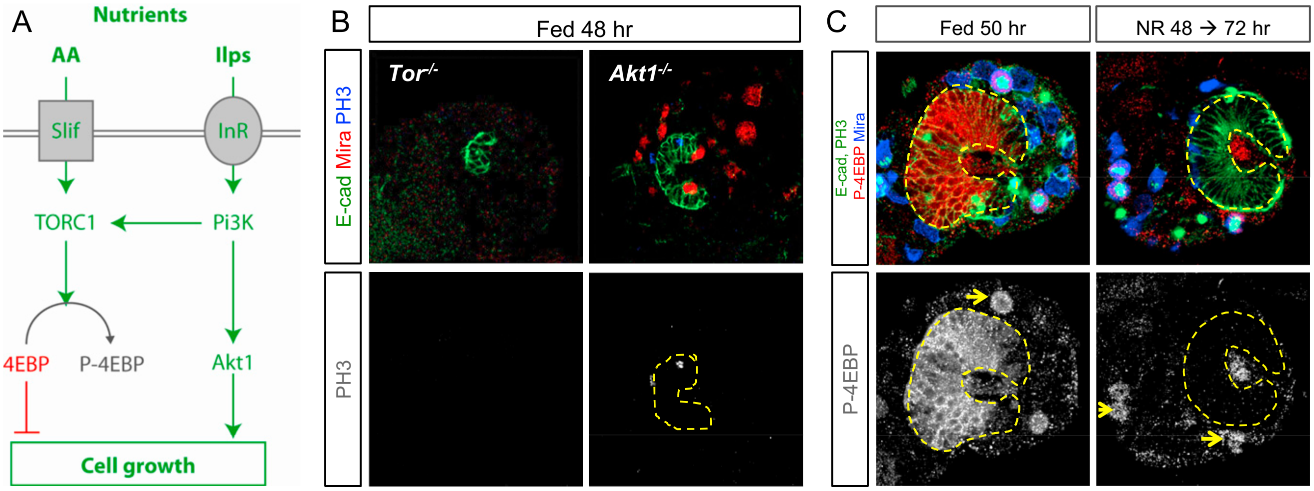

Figure S2. TOR/InR/Pi3K Signaling Promotes NE Expansion during Early Larval Stages, Related to Results

(A) Simplified schematic representation of the TOR/InR/Pi3K network. In cells, amino-acid sensors respond to dietary nutrients by activating the TOR kinase, which phosphorylate 4E-BP to promote RNA translation. Organismal growth is regulated by Ilps that bind the InR to activate Pi3K and Akt1. Both pathways converge in promoting cell growth. The TOR kinase is a central node for nutrient sensing and cell growth activation.

(B) The NE of *Tor*$^{\Delta P}$ mutant larvae fails to initiate proliferation. In *Akt1*^{1} mutants, NE expansion is also severely affected. E-cad (green), Mira (red), PH3 (white).

(C) In the expanding NE of 50 hr larvae, 4E-BP is strongly phosphorylated. However, if the larvae is transferred to NR conditions for 24 hr, phosphorylated 4E-BP becomes undetectable in the NE, while still present in some central brain PH3+ neuroblasts (yellow arrows). The medulla is delineated by yellow dashed line. E-Cadherin and PH3 (green), p-4E-BP (red), Mira (blue).

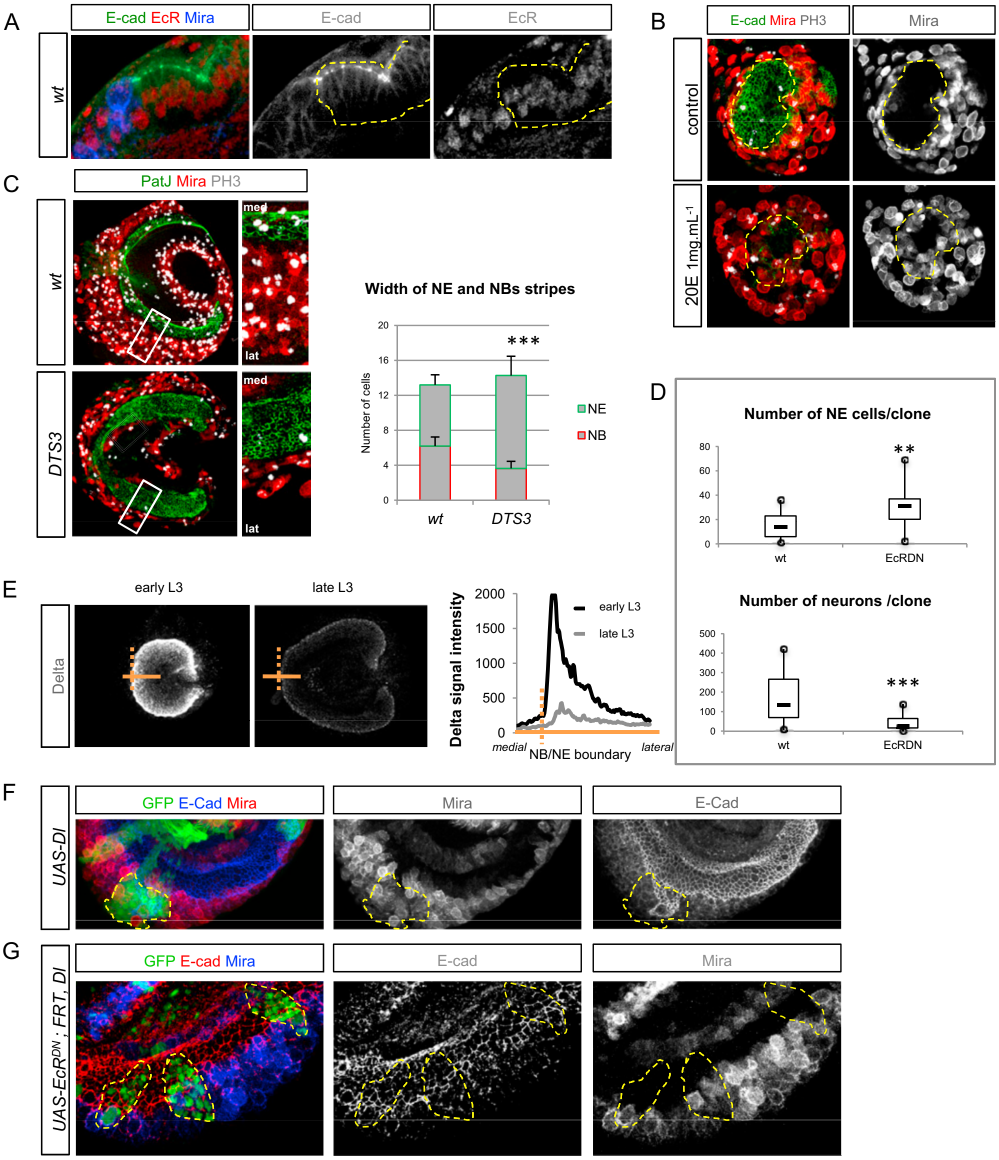

Figure S3. Ecdysone Signaling Regulates Progenitor Pool Size via Delta, Related to Figure 4

(A) Frontal section through the medulla of a 96 hr larvae showing that NE cells (E-cad in green), express EcR (in red).

(B) The NE of early L3 CNSs explanted for 24 hr in a culture medium containing 1mg/mL of 20E (20-hydroxyecdysone) is almost entirely converted in neuroblasts. In the control medium (1μg/ml of 20E), the NE continues dividing. E-cad (green), Mira (red), PH3 (white).

(C) *mld*^{DTS3} is a temperature sensitive an allele of the zinc finger *molting defective* (*mld*) gene required for ecdysone biosynthesis in the prothoracic gland (Neubueser et al., 2005). A shift to the restrictive temperature (29°C) at early L3 abrogates the late-larval ecdysone pulse, allowing *DTS3* mutant larvae to wander without pupariating for up to 15 days (Holden et al., 1986). The NE of *mld*^{DTS3} larvae, switched to restrictive temperatures for 6 days, is larger than the NE of *wt* late L3 larvae. Conversely, fewer NBs are produced in *mld*^{DTS3} mutants. E-cad (green), Mira (red), PH3 (white). The associated histogram depicts the average width of NE and medulla NB stripes in *wt* wandering L3 and *mld*^{DTS3} mutant larvae. The NE width is measured for the anterior half of the NE, and only NE cells located medial to the lamina furrow on the lateral side of the NE are taken in account. *wt* NE (n = 10, mean = 7.0, SD = 1.1), *wt* NB (n = 10, mean = 6.2, SD = 1); *DTS3* NE (n = 11, mean = 10.6, SD = 2.2). *DTS3* NB (n = 11, mean = 3.6 SD = 0.8). ***p < 0.001.

(D) Plots showing that the number of NE cells is increased in *EcR*^{DN} clones compared to *wt*. wt (mean = 14.7, n = 25, SD = 9); *EcR*^{DN} (mean = 29.5, n = 14, SD = 18). **p < 0.001. Plots showing that the number of neurons cells decreases by ~70% in *EcR*^{DN} clones compared to *wt*. *wt* (mean = 170, n = 17, SD = 134); *EcR*^{DN} (mean = 45, n = 12, SD = 48). ***p < 0.001.

(E) Plot depicting the signal intensity of Delta in early and late NEs along a medial-to-lateral axis (orange line). Note that for both early and late stages, immunostaining and image acquisition were performed under the same conditions.

(F) Clones misexpressing Delta delays NE-to-NB conversion. E-cad (blue), Mira (red).

(G) Loss of Delta in EcR^{DN} clones abrogates the delay in the progression of the proneural wave. GFP (green), E-cad (red) and Mira (blue).

Cell PRESS Open ACCESS

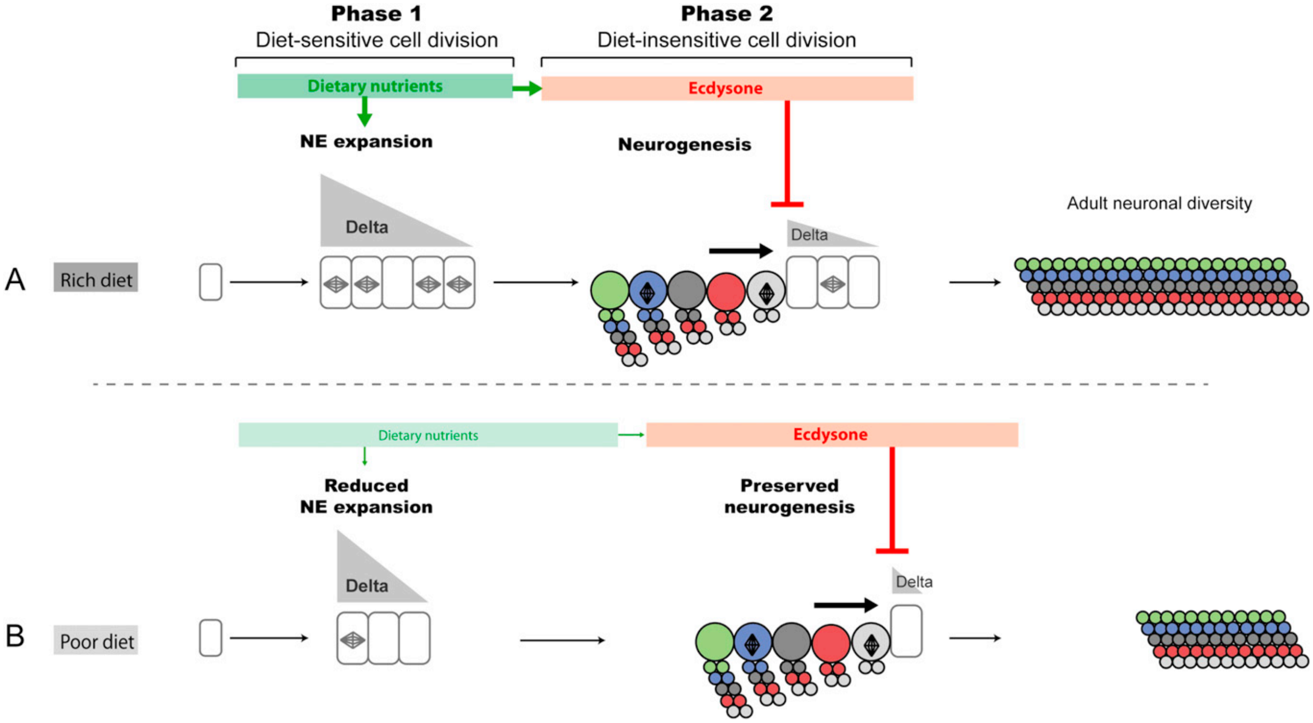

Figure S4. A Strategy for Preserving Neuronal Diversity in the Medulla When Neuron Numbers Are Reduced by Suboptimal Nutrition, Related to Results

(A) The NE undergoes a phase of expansion (phase 1) and a phase of conversion into neurogenic NBs (phase 2). NE expansion is promoted by nutrients through the TOR/InR/PI3K network, and is terminated by ecdysone that promotes NE-to-NB conversion during late larval stages (after 60 hr), through the downregulation of Delta. In contrast to phase 1, neural progenitor division during phase 2 is largely diet-insensitive ensuring that NBs generate their full repertoire of neurons independently of nutritional conditions.

(B) Under suboptimal nutritional conditions (poor diet), NE expansion is impaired during phase 1, leading to a reduced neural progenitor pool by the end of larval stages. However, medulla neuroblasts, which are produced during the diet-insensitive phase 2, remain able to generate their normal set of progeny. Consequently, the neuron numbers in the brain are reduced but the diversity is preserved.
